# Supplementary material for: Detection of Ovarian Cancer through Exhaled Breath by Electronic Nose: A Prospective Study
Source: Cancers (Basel). 2020 Aug 25;12(9):2408. doi: 10.3390/cancers12092408 (PMC7565069; doi:10.3390/cancers12092408)
Supplement: Supplementary file 1 [file cancers-12-02408-s001.pdf]

Supplementary Materials: Detection of Ovarian Cancer through Exhaled Breath by Electronic Nose: A Prospective Study

Francesco Raspagliesi, Giorgio Bogani, Simona Benedetti, Silvia Grassi, Stefano Ferla and Susanna Buratti

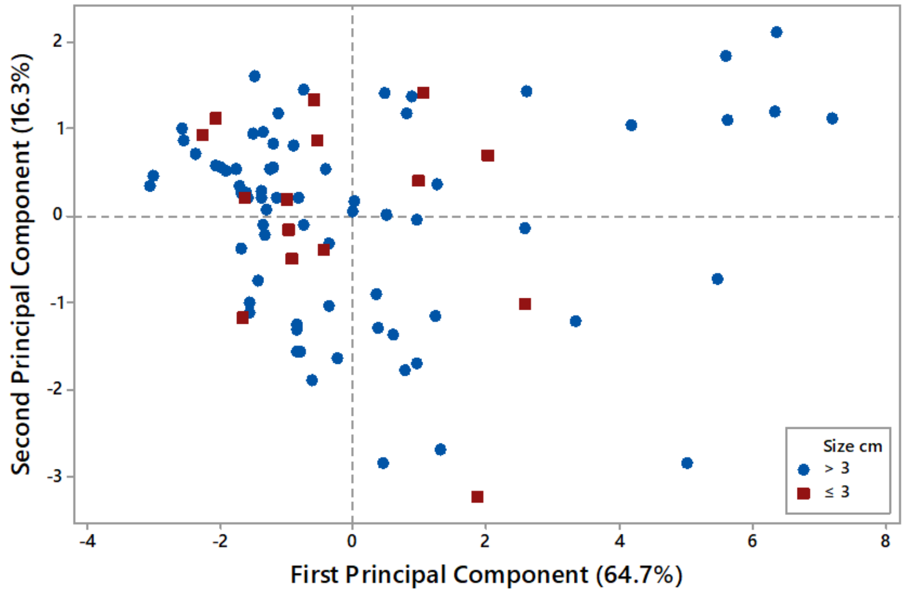

Figure S1. The impact of tumor size on e-nose signals. Legend to figure: PCA (principal component analysis) score plot of electronic nose data collected on OC cases on the basis of tumor size: major to 3 cm (blue) and minor or equal to 3 (red).

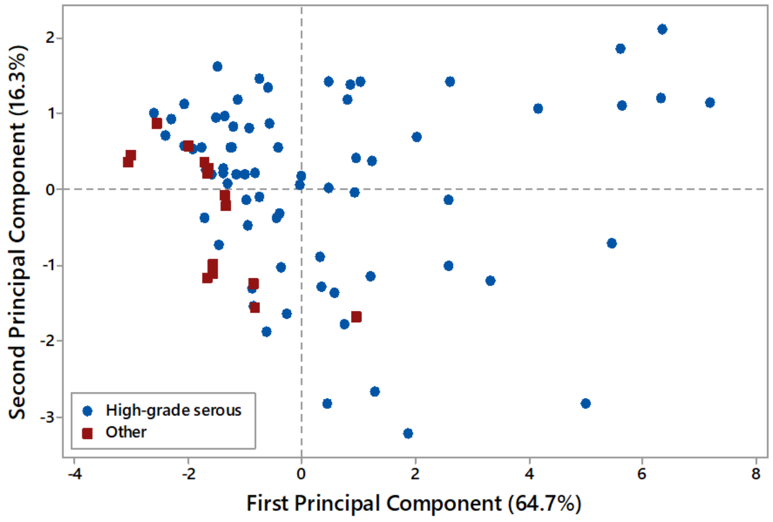

Figure S2. The impact of histology on e-nose signals.Legend to figure: The PCA (principal component analysis) score plot of electronic nose data collected on high-grade serous (blue) and other histology (red) samples collected from OC patients. Patients with other histology (other than high grade serous) are clustered in the left corner of the whole OC group.

Table S1. Baseline patients' characteristics.

**Characteristic**	**A) Ovarian Early Stage Malignances (*n* = 32)**	**B) Ovarian Late Stage Malignances (*n* = 54)**	**C) Benign Lesions (*n* = 51)**	**D) Healthy Subjects (*n* = 114)**	***P value**<br>*A-D*<br>*B-D*<br>A-C<br>B-C**
Age, mean (SD)	56.75 (±15.1)	59.4 (±13.6)	52.6 (±14.8)	41.8 (±13.5)	**0.0001**<br>**0.0001**<br>0.221<br>**0.015**
Menopause (%)	20 (62.5)	38 (70)	28 (54.9)	38 (33.3)	**0.003**<br>**0,001**<br>0,495<br>0,101
BMI, mean ± SD	24.9 (±5.4)	23.9 (±4.9)	24 (±4.4)	23.4 (±4.9)	0.13<br>0.53<br>0.40<br>0.91
Smoking patients, N (%)	10 (31.2)	5 (9.2)	11 (21.5)	28 (24.5)	0.44<br>**0.01**<br>0.32<br>0.07
CA125, mean (SD)	270 (±108)	2061.1 (±1014)	50.6 (±79.4)	21 (±8.3)	**< 0.0001**<br>**< 0.0001**<br>**< 0.0001**<br>**< 0.0001**
Surgery, N (%)	32 (100)	54 (100)	44 (86.3)	0	**< 0.001**<br>**< 0.001**<br>**< 0.028**<br>**< 0.005**
No surgical treatment, N (%)	0 (0)	0 (0)	7 (13.7)	114 (100)	**< 0.001**<br>**< 0.001**<br>**< 0.028**<br>**< 0.005**

Abbreviations: BMI, body mass index; SD, standard deviation; CA125: Carbohydrate Antigen 125; *Variables characterized by p value <0.05 have been considered statistically significant and presented in bold characters.

Table S2. Final histological diagnosis for patients with adnexal masses who had surgery.

**Histological subtype**	**Number (Percentage)**
**Ovarian Malignancies (*N*=86)**	
**Histologies**	
High grade serous carcinoma, *N* (%)	63 (71.5)
Endometrioid adenocarcinoma, *N* (%)	2 (2.3)
Mucinous carcinoma, *N* (%)	5 (5.6)
Clear cell carcinoma, *N* (%)	1 (1.1)
Ovarian carcinosarcoma, *N* (%)	2 (2.3)
Low grade serous carcinoma, *N* (%)	8 (9)
Steroid cell tumor, *N* (%)	1 (1.1)
Granulosa cell tumor, *N* (%)	1 (1.1)
Immature teratoma, *N* (%)	1 (1.1)
Malignan Brenner carcinoma, *N* (%)	2 (2.3)
**Median (range) size of ovarian mass, cm**	
- Early stage disease (FIGO stage I-II)	5 (2–10)
- Advanced stage disease (FIGO stage III)	6 (3–15)
**Stage at presentation**	
FIGO stage I	25 (29.1)
Stage IA	8 (9.3)
Stage IB	0
Stage IC	17 (19.7)
FIGO stage II	7 (8.1)
Stage IIA	5 (5.8)

Stage IIB	2 (2.3)
FIGO stage III	54 (62.8)
Stage IIIA	1 (1.1)
Stage IIIB	4 (4.6)
Stage IIIC	49 (56.9)
**Benign Neoplastic Ovarian Cysts (*N*=51)**	
Median (range) of ovarian mass, cm	7 (5–20)
No surgery, *N* (%)	7 (14)
Mucinous cystadenoma, *N* (%)	1 (2)
Serous cystadenoma, *N* (%)	5 (10)
Serous cystadenofibroma, *N* (%)	6 (12)
Cystadenofibroma, *N* (%)	2 (4)
Fibroma, *N* (%)	9 (18)
Serous adenofibroma, *N* (%)	3 (6)
Endometrioid adenofibroma, *N* (%)	3 (6)
Endometrioma, *N* (%)	8 (16)
Mature cystic teratoma, *N* (%)	2 (4)
Paratubaric cyst, *N* (%)	3 (6)
Chronic follicular salpingitis, *N* (%)	2 (4)

Table S3. Generic confusion matrix identifying true positive (TP), false positive (FP), true negative (TN), false negative (FN) and the reference equation to calculate sensitivity (SENS) and specificity (SPEC).

		**Condition as Determined by the Reference Test**		
		**Positive**	**Negative**	**Column Total**
Outcome of the diagnostic test	Positive	True positive (TP)	False positive (FP)	TP + FP<br>Total number of subjects positive test
	Negative	False Negative (FN)	True negative (TN)	FN + TN<br>Total number of subjects negative test
	Column total	TP + FN<br>Total number of subjects with given condition	FP + TN<br>Total number of subjects without given condition	N = TP + TN + FP +FN<br>Total number of subjects in study
Figures of merit	**Sensitivity = TP/(TP+FN)**			
	**Specificity = TN/(TN+FP)**			
	**Accuracy = (TN + TP)/(TN + TP + FN + FP)**			

© 2020 by the authors. Submitted for possible open access publication under the terms and conditions of the Creative Commons Attribution (CC BY) license (http://creativecommons.org/licenses/by/4.0/).
